# Exploring the clinical features of narcolepsy type 1 versus narcolepsy type 2 from European Narcolepsy Network database with machine learning

**DOI:** 10.1038/s41598-018-28840-w

**Published:** 2018-07-13

**Authors:** Zhongxing Zhang, Geert Mayer, Yves Dauvilliers, Giuseppe Plazzi, Fabio Pizza, Rolf Fronczek, Joan Santamaria, Markku Partinen, Sebastiaan Overeem, Rosa Peraita-Adrados, Antonio Martins da Silva, Karel Sonka, Rafael del Rio-Villegas, Raphael Heinzer, Aleksandra Wierzbicka, Peter Young, Birgit Högl, Claudio L. Bassetti, Mauro Manconi, Eva Feketeova, Johannes Mathis, Teresa Paiva, Francesca Canellas, Michel Lecendreux, Christian R. Baumann, Lucie Barateau, Carole Pesenti, Elena Antelmi, Carles Gaig, Alex Iranzo, Laura Lillo-Triguero, Pablo Medrano-Martínez, José Haba-Rubio, Corina Gorban, Gianina Luca, Gert Jan Lammers, Ramin Khatami

**Affiliations:** 1 Center for Sleep Medicine, Sleep Research and Epileptology, Klinik Barmelweid AG, Barmelweid, Switzerland; 2 Neurology Department, Hephata Klinik, Schwalmstadt, Germany; 3 Centre de Reference Nationale Maladies Rares, Narcolepsie et Hypersomnie Idiopathique, Service Neurologie, Hôpital Gui-de-Chauliac, INSERM U1061 Montpellier, France; 4 Department of Biomedical and Neuromotor Sciences (DIBINEM), Alma Mater Studiorum, University of Bologna, Bologna, Italy; 5 IRCCS Istituto delle Scienze Neurologiche di Bologna, ASL di Bologna, Bologna, Italy; 6 Department of Neurology and Clinical Neurophysiology, Leiden University Medical Center, Leiden, The Netherlands; 7 Neurology Service, Multidisciplinary Sleep Unit, Hospital Clínic of Barcelona, IDIBAPS, CIBERNED, Barcelona, Spain; 8 Helsinki Sleep Clinic, Vitalmed Research Center, Helsinki, Finland; 9 Sleep Medicine Center Kempenhaeghe, Heeze, The Netherlands; 10 Eindhoven University of Technology, Eindhoven, The Netherlands; 11 Sleep and Epilepsy Unit, Clinical Neurophysiology Department, Gregorio Marañón University Hospital, Complutense University of Madrid (UCM), Madrid, Spain; 12 Serviço de Neurofisiologia, Hospital Santo António/Centro Hospitalar do Porto and Instituto Ciências Biomédicas Abel Salazar, Universidade do Porto, Porto, Portugal; 13 Neurology Department and Centre of Clinical Neurosciences, First Faculty of Medicine, Charles University and General University Hospital, Prague, Czech Republic; 14 Unidad de Neurofisiología y Trastornos del Sueño. Hospital Vithas Internacional Madrid, Madrid, Spain; 15 Center for Investigation and Research in Sleep, Lausanne University Hospital, Lausanne, Switzerland; 16 Department of Clinical Neurophysiology, Institute of Psychiatry and Neurology, Warsaw, Poland; 17 Department of Sleep Medicine and Neuromuscular Disorders, University of Münster, Münster, Germany; 18 Neurology Department, Sleep Disorders Clinic, Medical University of Innsbruck, Innsbruck, Austria; 19 Sleep and Epilepsy Center, Neurocenter of Southern Switzerland, Lugano, Switzerland; 20 Department of Neurology, Inselspital, Bern University Hospital, and University of Bern, Bern, Switzerland; 21 Neurology Department, Medical Faculty of P. J. Safarik University, University Hospital of L. Pasteur Kosice, Kosice, Slovak Republic; 22 Institute of Molecular Medicine Portugal, Medical Faculty Lisbon University, Lisbon, Portugal; 23 Fundació Institut d’Investigació Sanitària Illes Balears (IdISBa), Hospital Universitari Son Espases, Palma de Mallorca, Spain; 24 AP-HP, Pediatric Sleep Center, CHU Robert-Debré, Paris, France; 25 National Reference Centre for Orphan Diseases, Narcolepsy, Idiopathic Hypersomnia and Kleine-Levin Syndrome (CNR narcolepsie-hypersomnie), Paris, France; 26 Neurology Department, University Hospital Zurich, Zurich, Switzerland; 27 Centre Neuchatelois de Psychiatrie, Neuchatel, Switzerland; 28 Sleep Wake Center SEIN Heemstede, Stichting Epilepsie Instellingen Nederland, Heemstede, The Netherlands

## Abstract

Narcolepsy is a rare life-long disease that exists in two forms, narcolepsy type-1 (NT1) or type-2 (NT2), but only NT1 is accepted as clearly defined entity. Both types of narcolepsies belong to the group of central hypersomnias (CH), a spectrum of poorly defined diseases with excessive daytime sleepiness as a core feature. Due to the considerable overlap of symptoms and the rarity of the diseases, it is difficult to identify distinct phenotypes of CH. Machine learning (ML) can help to identify phenotypes as it learns to recognize clinical features invisible for humans. Here we apply ML to data from the huge European Narcolepsy Network (EU-NN) that contains hundreds of mixed features of narcolepsy making it difficult to analyze with classical statistics. Stochastic gradient boosting, a supervised learning model with built-in feature selection, results in high performances in testing set. While cataplexy features are recognized as the most influential predictors, machine find additional features, e.g. mean rapid-eye-movement sleep latency of multiple sleep latency test contributes to classify NT1 and NT2 as confirmed by classical statistical analysis. Our results suggest ML can identify features of CH on machine scale from complex databases, thus providing ‘ideas’ and promising candidates for future diagnostic classifications.

## Introduction

Narcolepsy is a rare central hypersomnia (CH) with an estimated prevalence of 0.02% in the European population^1^. The diagnosis of narcolepsy is challenging for several reasons. First, excessive daytime sleepiness (EDS), the key feature of narcolepsy, is shared by many other central hypersomnias and also a prevalent life-style consequence of insufficient sleep in our modern societies. Second, various forms of narcolepsy exist and only in narcolepsy type-1 (NT1, formerly referred as narcolepsy with cataplexy) low or absent cerebrospinal fluid (CSF) hypocretin levels serve as a specific biomarker^2–4^. In contrast, in narcolepsy type-2 (NT2) or other variants of central hypersomnias (i.e. idiopathic hypersomnia or hypersomnias associated with psychiatric diseases), specific biomarkers are absent^5^. Third, the current international diagnostic criteria are based mainly on clinical experience but large multicenter international data are still missing. Even though international diagnostic criteria have been published, they may not be used in all countries. For example, CSF-hypocretin measurement is not available in many centers.

Therefore, the European Narcolepsy Network (EU-NN), an association of leading European sleep centers, launched the first prospective European web-based database for narcolepsy and related disorders which allows collection, storage and dissemination of data on narcolepsy in a comprehensive and systematic way^1^. Currently this database includes 317 mixed types of variables per patient (e.g., categorical variables and discrete/continuous numeric variables from questionnaire data like features and history of cataplexy, laboratory data of multiple sleep latency test (MSLT) and polysomnography (PSG), biomarkers like CSF hypocretin levels and HLA DQB1*06:02) and 1380 patients from 26 centers with a number of missing data and multicollinearity, making it difficult to analyze with conventional analytics methods.

One major goal of the EU-NN database is to identify distinctive phenotypes of central hypersomnias and to identify new biomarkers specific for these phenotypes. Machine learning (ML) tools are becoming increasingly popular in medicine as these methods are able to detect patterns of symptoms and unveil information that is not visible for humans. Unlike human knowledge machines will extract their own information based on an iterative information processing. ML is specifically powerful in achieving classifiers (i.e. NT1 vs NT2) when extracting hidden information from big and disparate data sources like the EU-NN database at machine scale, i.e., it frees from the limitations of human scale thinking. Boosting is one of the most powerful ML methods for selecting features and weight their predictive contribution to the classifier. It combines the outputs of many weak learners to produce a powerful “committee”^6,7^. The predictions from all of the weak learners are combined through a weighted “majority vote” to produce the final prediction^7^. As a decision tree-based ensemble method, boosting can naturally incorporate mixtures of numeric and categorical predictor variables and missing values^7^. It can reduce multicollinearity and overfitting (overfitting is a common problem in ML which means that the model can fit the training data well but cannot generalize to data outside the training set, such as the data in the testing set or data from new cases) problems through shrinkage strategy^7^, making it more resistant to multicollinearity than other ML methods like neural network^8^. The merits of boosting may render it a suitable and powerful algorithm to model the EU-NN database for empirical classification of narcolepsy.

In this data-driven study we use boosting to explore the predictive features of narcolepsy NT1 and NT2 from EU-NN database. We choose Stochastic Gradient Boosting (SGB) model, an advanced supervised learning (i.e., the instances are given with known labels which are NT1 and NT2 in this study) method in the boosting algorithm family that usually outperforms others^9,10^ as it combines boosting with bootstrap averaging^10^. SGB model has good interpretability, i.e., it can identify the variable importance^11^. The resulting relative variable influences can be used for feature selection. Therefore, SGB is a model with built-in feature selection, which is more efficient than the usual feature selection procedures (i.e., wrapper methods and filter methods) in which the searching routine for the right predictors is external to the model^12^. To do the feature selection with SGB, we first construct a SGB model for the classification of NT1 and NT2. Next we evaluate the performances of the built SGB model in the testing set. The model correctly ‘learns’ the clinical features of NT1 and NT2 if it gives good performances. By checking the variable importance ranked by the model, machine could feed us back with clinical features of narcolepsy on machine scale (i.e., machine tells human how it ranks the mixed features of narcolepsy in EU-NN database).

## Results

### The optimal model classifying NT1 and NT2

**Figure 1:**
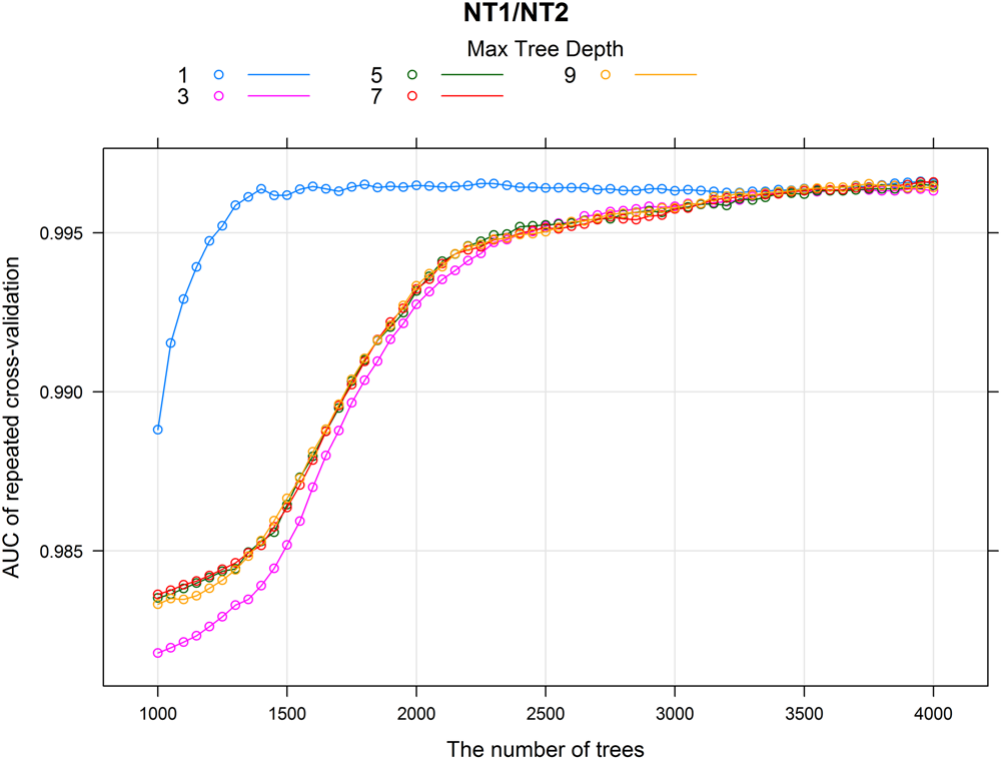
The results of the performance of cross-validation. The colored lines indicate different interaction depths (i.e., maximal tree depths). Each data point in the figure represents one classifier. For example, the magenta data point at (1500, 0.985) indicates a model built with 1500 trees and the tree depth is 3, and this model gives an AUC value of 0.985 in the 10-fold cross-validation with 10 times repeats.

The optimal classifier is constructed with 3950 trees (tree depth is 1). While any combination of the number of trees and tree depth can build a model with high value of area under the Receiver Operating Curves (ROC) (i.e., AUC) (Fig. 1), the optimal model obtains high predictive performances on the testing set, i.e., the accuracy of prediction is 0.9943 (95% confidence interval [CI]: 0.9686–0.9999), AUC is 0.999, Kappa is 0.9778, and sensitivity and specificity are 0.9933 and 1, respectively. Only one case is not correctly predicted by the classifier, a female diagnosed as NT1 by the clinician but classified as NT2 by the SGB model. After revisiting her data we find it far more accurate to classify the patient as NT2 instead of NT1 because of the absence of cataplexy and missing value of hypocretin level, suggesting that the prediction of our SGB classifier is correct.

Our model can give the probability of each prediction at an individual level and thus provide insights into the learned rules of SGB classification. Table 1 shows examples that SGB classification for each patient is based on a numeric probability for NT1 and NT2, respectively. For example, one patient in the testing set who has undetectable CSF hypocretin levels (below 40 pg/ml) but no history of cataplexy should be diagnosed as NT1 according to our knowledge of hypocretin deficit. SGB calculates a probability of being NT1 and NT2 of this patient of 57.2% and 42.8%, respectively suggesting that our model indeed takes both cataplexy and hypocretin level into account to make final prediction.

**Table 1:** The example of predictive probability of classifier NT1/NT2 on the testing set.

The number of patient	Probability of NT1	Probability of NT2
1	20.2%	79.8%
2	17.5%	82.5%
3 :	15.2% :	84.8% :

### Feature selection of the optimal model

**Figure 2:**
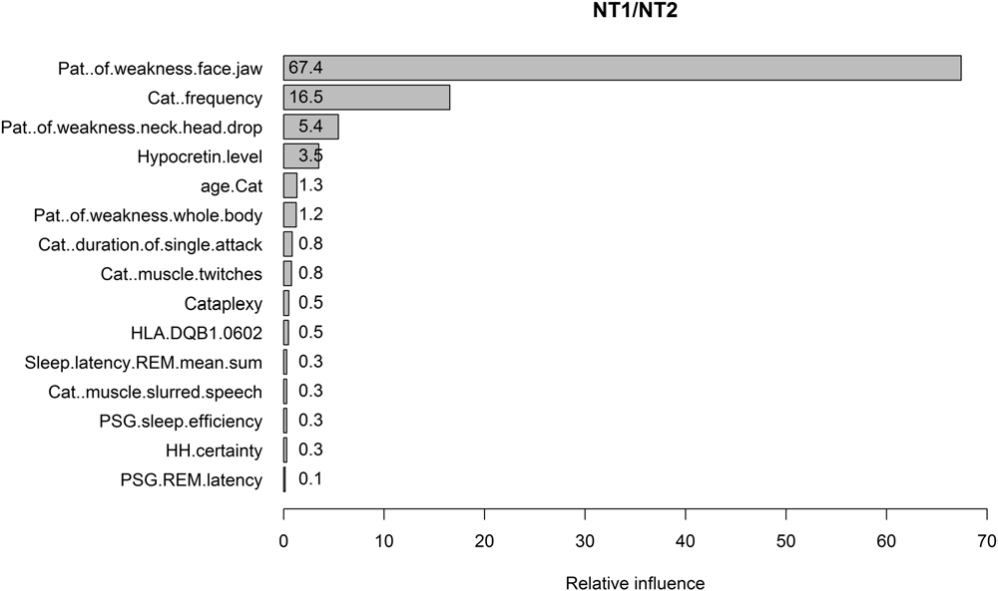
The relative influences of predictors in the classifiers of NT1/NT2. The variable names written on the vertical axis are the ones giving relative influence larger than 0.1. “Pat.” is short for pattern, “Cat.” is cataplexy. “Sleep.latency.REM.mean.sum” is the mean REM sleep latency of multiple sleep latency test. “HH.certainty” is the clinical certainty of hypnagogic hallucinations.

Feature selection results in a ranking of 35 predictors that contribute to the classification of NT1 and NT2 (see Supplementary Figure 1). 15 predictors yield relative influence (RI) larger than 0.1 (Fig. 2). The most influential predictors are the ones related to cataplexy features whereas hypocretin level contributes relatively less to the model, although it ranks as No. 4. In addition, “PSG.sleep.efficiency” and “HLA.DQB1.0602” contribute to the prediction. Wilcoxon rank sum test shows that the median of PSG sleep efficiency is significantly different between patients with NT1 and NT2 (86% [79.7–91.3%] vs. 94% [89.8–96%], P < 0.001). Fisher’s exact test shows that the odd ratio (OR) of HLA DQB1*06:02 -positive/HLA DQB1*06:02 -negative is significantly larger than 1 in NT1/NT2 (OR = 18.8, 95% CI: 9.54–38.11, P < 0.001), indicating that HLA DQB1*06:02 positive is more likely to occur in NT1.

Mean REM sleep latency of MSLT (“Sleep.latency.REM.mean.sum”), REM sleep latency of PSG (“PSG.REM.latency”), and hypnagogic hallucinations (“HH.certainty”) are three predictors contributing to the model. Welch two sample t-test and Wilcoxon rank sum test show that the mean (5.68 ± 0.20 min vs. 8.25 ± 0.49 min, P < 0.001) and the median (4.8 [2.8–7.7] min vs. 8 [5.8–11] min, P < 0.001) of the mean REM sleep latency of MSLT are significantly different between patients with NT1 and NT2. The median (14 [4–79] min vs. 59 [8.25–77] min, P = 0.009) rather than the mean (51.99 ± 3.77 min vs. 58.33 ± 6.01 min, P = 0.37) of the PSG REM sleep latency is significantly different. Fisher’s exact test finds weak differences in the odd of HH.certainty-definite/HH.certainty-indefinite between NT1 and NT2 (OR = 2.38, 95% CI: 0.95–5.62, P = 0.046).

### The mean REM sleep latency of MSLT

Our finding that mean REM sleep latency contributes to the classification of NT1 and NT2 is unexpected, as only the number of sleep-onset REM sleep period (SOREMP) is a well-known disgnostic marker for narcolepsy^13^. We then perform logistic regression with NT1 and NT2 as dependent variable and the number of SOREMP and the mean REM sleep latency of MSLT as explanatory variables, to confirm the findings of the machine. Complete MSLT datasets are available from 321 NT1 and 83 NT2 patients to build the model. The model passes the Hosmer-Lemeshow goodness of fit test (P = 0.796). Both the mean REM sleep latency and the number of SOREMP are strongly significant predictors (P < 0.001 for both variables) to classify NT1 and NT2, and the ORs are 0.89 (95% CI: 0.84–0.95) and 1.85 (95% CI: 1.45–2.38), respectively. These results suggest that the decrement of 1-min mean REM sleep latency increases the odd of being NT1 by a factor of 1.12 (i.e., 1/0.89 = 1.12) and the increment of 1 SOREMP results in an increment in the odd of being NT1 by a factor of 1.85. The one unit increment in SOREMP can induce larger increase in the odd of being NT1; however, the mean REM sleep latency may be a more precise variable because of its narrower CI.

Using ROC analysis, we recognize that mean REM sleep latency or the number of SOREMP alone cannot yield a good classifier for NT1 and NT2. AUC values between 0.7 and 0.8 are considered as fair. Mean REM sleep latency alone achieves an AUC value of 0.67 (sensitivity/specificity/positive predictive value (PPV)/negative predictive value (NPV): 56.7%/72.3%/69.8%/11.2%). Similarly, the number of SOREMP alone obtains a value of 0.69 (sensitivity/specificity/PPV/NPV: 58.6%/71.3%/67.1%/12.2%). However, the classifier combining both variables results in a fair classifier of AUC 0.75 (sensitivity/specificity/PPV/NPV: 65.1%/74.7%/64.4%/9.1%). The optimal cut-off point is 5.6 min for the mean REM sleep latency, i.e., patients with a mean REM sleep latency of MSLT shorter than 6-min may have relative higher probability to be diagnosed as NT1.

**Figure 3:**
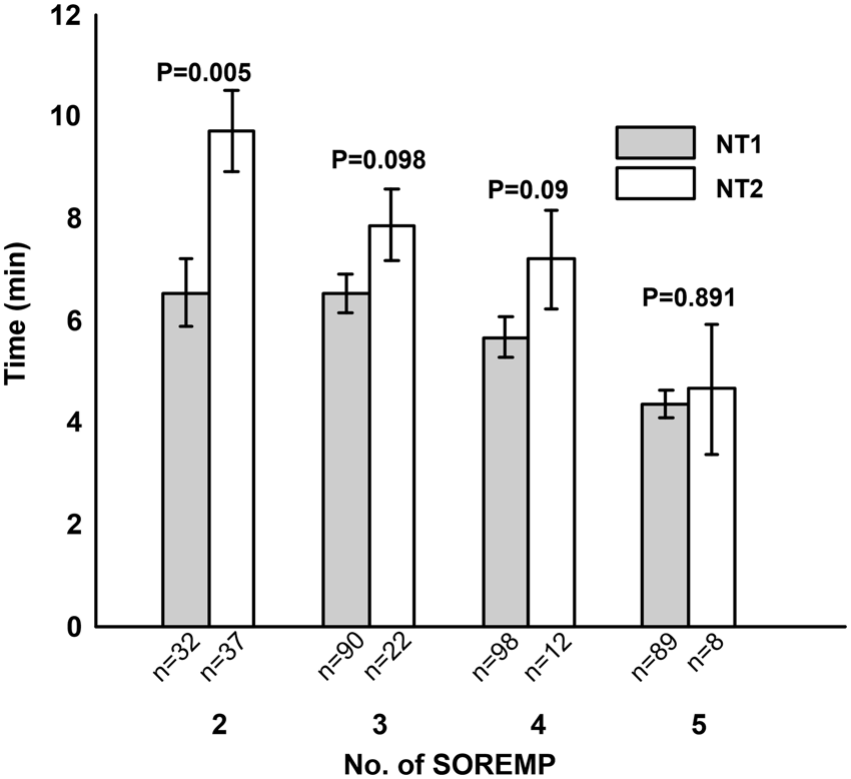
Comparisons of mean REM sleep latency between NT1/NT2 patients with the same number of SOREMP. The number of patient of each subgroup is given on the x-axis. P-values are given by Wilcoxon rank sum tests. Please note the error bars are the ones of standard error of mean. Considering the small sample size of NT2 patients, the distributions of the mean REM sleep latency in NT2 patients with 4 and 5 SOREMP may hardly fit normal distribution. So we provide the results of Wilcoxon rank sum test which tests the median rather than the mean of compared groups in the figure.

Subgroup comparison is a simple and direct way to illustrate that mean REM sleep latency is useful in classifying NT1 and NT2 patients when they have the same number of SOREMP. The results are shown in Fig. 3. Wilcoxon rank sum test indicates that the medians of the mean REM sleep latency are significantly different in NT1 and NT2 patients with 2 SOREMPs (P = 0.005), and it tends to be statistically different in NT1 and NT2 patients with 3 and 4 SOREMPs (P = 0.098 and 0.09, respectively).

We further correlate mean MSLT REM sleep latency to other biomarkers like hypocretin levels and HLA DQB1*06:02, because it may provide insights into pathophysiology of REM sleep propensity in narcolepsy. Totally 157 patients (143 NT1 and 14 NT2) have complete MSLT and hypocretin measurements. The mean REM sleep latency of MSLT significantly correlates to the hypocretin levels (n = 157, correlation coefficient r = 0.37, P < 0.001). This correlation is seen in patients with NT1 (n = 143, r = 0.18, P = 0.03) but not in patients with NT2 (n = 14, r = 0.319, P = 0.266), although latter result should be interpreted with caution due to small sample size of NT2. When dividing these 157 patients into 2 subgroups with the cut-off of 5.6 min of mean REM sleep latency (i.e., the mean REM sleep latency <5.6 min in 77 patients, and >5.6 min in 80 patients), we find significant lower hypocretin levels in the first group (22.75 ± 5.55 vs. 83.11 ± 12.52, both Welch two sample t-test and Wilcoxon rank sum test give P < 0.001). These results indicate that shorter mean REM sleep latency are associated with lower hypocretin levels.

Similarly, we also test the relationship between the mean REM sleep latency and HLA DQB1*06:02. Totally 285 patients (247 NT1 and 38 NT2) with complete measurements of MSLT and HLA DQB1*06:02 are divided into two subgroups (154 patients with mean REM sleep latency <5.6 min and 131 patients with mean REM sleep latency >5.6 min). Fisher’s exact test shows that OR of HLA DQB1*06:02-positive/HLA DQB1*06:02-negative is significantly larger than 1 in the group with shorter mean REM sleep latency (OR = 3.87, 95% CI: 1.60–10.40, P = 0.001), indicating that HLA DQB1*06:02 positivity is more likely to occur in patients with shorter mean REM sleep latency.

### Models constructed without cataplexy features and hypocretin

**Figure 4:**
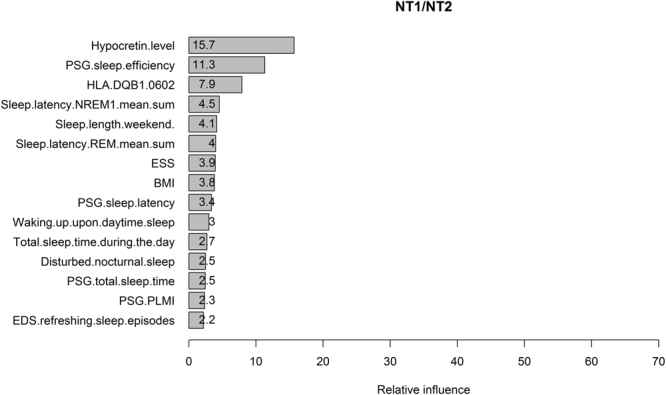
The relative influences of the features contributing to the classification of NT1/NT2 selected by the SGB model built without cataplexy features. “Sleep.latency.NREM1.mean.sum” is the mean non-REM stage 1 sleep latency of MSLT. ESS is Epworth sleepiness scale. “Waking.up.upon.daytime.sleep” means wake up from daytime sleep. PLMI is periodic limb movement index. “EDS.refreshing.sleep.episodes” means excessive daytime sleepiness (EDS) is refreshed after sleep episodes.

Approximately half of the influential predictors in Fig. 2 are already embedded in diagnostic criteria for NT1 or NT2. Although this finding confirms that our SGB algorithm has correctly learned the diagnostic criteria made by humans, it may hinder us to further detect new useful features to classify narcolepsy. We therefore build a new model excluding cataplexy features (built with 4000 trees and tree depth of 5). The top 25% features and their relative contributions are shown in Fig. 4. The total relative influences of these 15 features are 74%. CSF hypocretin level is the most contributing feature followed by PSG sleep efficiency and the presence of HLA DQB1*06:02. Although these results are plausible and consistent with existing knowledge, we suggest to interpret the results carefully due to the low performances of the model (in testing set, the AUC = 0.874, Kappa = 0.304, accuracy = 0.874, sensitivity = 0.987, specificity = 0.231). The values of AUC and accuracy are still good, but the dramatic decrease in Kappa (0.9778 vs. 0.304) and specificity (1 vs. 0.231) indicate that the high AUC and accuracy are mainly caused by the imbalanced dataset (i.e., 149 NT1 vs. 26 NT2 patients in the testing set). So even a model predicting all the patients as NT1 can yield an accuracy of 0.85 and a sensitivity of 1 but a specificity of 0. In this case, Kappa and the balanced accuracy (i.e., the mean of sensitivity and specificity which is 0.609 here) are better metrics to evaluate our new model as a poor classifier for NT1 and NT2. We propose to consider the selected features as reliable only when the models have good performances.

**Figure 5:**
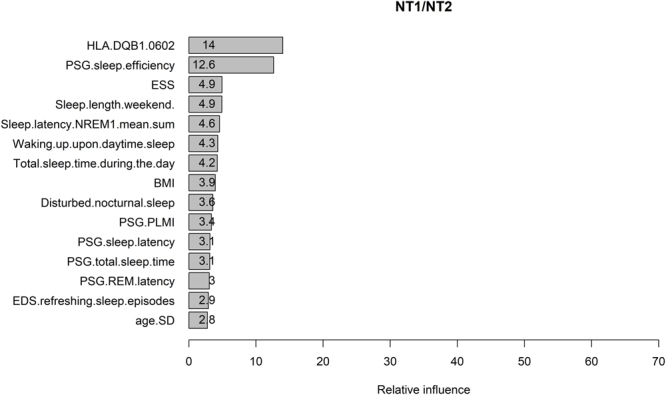
The relative influences of the features contributing to the classification of NT1/NT2 selected by the SGB model built without cataplexy features and hypocretin level. Please refer to figure legends of Fig. 3 and 4 for the names of the predictors. “age.SD” here means the age of sleep diagnosis.

Next we remove hypocretin level and update our model. The performance of the updated model (built with 3950 trees and tree depth of 5) further decreases (Kappa = 0.272, specificity = 0.192, sensitivity = 0.993, AUC = 0.843, accuracy = 0.874, balanced accuracy = 0.593). The ranking of the first 15 features selected are shown in Fig. 5. Their total influences are 75.4%. HLA DQB1*06:02 and PSG sleep efficiency are the most influential features. As already mentioned, the interpretation of the model should be cautious because of its poor performance.

Considering that the same cluster of predictors is selected by both models in Fig. 4 and 5, we analyze those predictors that are not included in Fig. 2 with traditional statistics. Welch Two Sample t-test reveals a larger Epworth Sleepiness Scale (ESS) in NT1 patients compared to NT2 (16.9 ± 0.16 vs. 15.2 ± 0.42, P < 0.001) and similar differences for BMI (27.7 ± 0.23 vs. 25.5 ± 0.44, P < 0.001) and longer daytime sleep (1.62 ± 0.07 h vs. 0.92 ± 0.10 h, P < 0.001). In MSLT NT1 patients have shorter mean non-REM stage 1 sleep latency (Sleep.latency.NREM1.mean.sum) (3.60 ± 0.14 min vs. 4.52 ± 0.42 min, Welch Two Sample t-test P = 0.038). In PSG measurements NT1 patients present with a higher periodic limb movement index (PLMI) (14.04 ± 1.31 vs. 5.68 ± 1.27, Welch Two Sample t-test P < 0.001) and shorter PSG sleep latency (4.35[2–8] min vs. 5.65[3–9] min, Wilcoxon rank sum test P = 0.025). NT1 patients report more frequently that they easily wake up from daytime sleep (OR of ‘easy to wake up upon daytime sleep/difficult or nearly impossible to wake up upon daytime sleep’ between NT1 and NT2 is 2.57, 95% CI: 1.45–4.52, P = 0.0009) and complain a disturbed nocturnal sleep (OR of ‘present disturbed nocturnal sleep/not present disturbed nocturnal sleep’ between NT1 and NT2 is 3.52, 95% CI: 2.23–5.61, P < 0.001). NT1 patients are more likely to feel refreshed after sleep than NT2 patients (OR of ‘EDS.refreshing.sleep.episodes-yes/EDS.refreshing.sleep.episodes-no or not always’ is 2.51, 95% CI: 1.44–4.42, P = 0.0007).

Remarkably, NT1 compared to NT2 patients report shorter sleep lengths during the weekend (Sleep.length.weekend) (7.97 ± 0.08 h vs. 9.06 ± 0.18 h, both Welch Two Sample t-test and Wilcoxon rank sum test result in a P < 0.001), whereas their differences in sleep lengths during the week are relatively small (7.36 ± 0.07 h vs. 7.64 ± 0.12 h; Welch Two Sample t-test P = 0.046 and Wilcoxon rank sum test P = 0.075). The differences of the sleep lengths between the weekend and the week are significantly longer in NT2 patients (NT1 vs. NT2: 0.64 ± 0.05 h vs. 1.41 ± 0.17 h, both Welch Two Sample t-test and Wilcoxon rank sum test give P < 0.001).

## Discussion

The goal of our study is to identify features of narcolepsy and weight their relative contributions to diagnosis from a huge and complex international multicenter database (EU-NN database). We use machine learning because unlike human knowledge and classical statistics, SGB will learn its own rules to classify NT1 and NT2 by taking the entire information of the database into account. In addition, SGB learns to select features independent from existing diagnostic classification and is thus relatively independent from human circular reasoning. Our results suggest that SGB is appropriate to classify narcolepsy subtypes with a high accuracy, and it can consider mixture of clinical features on machine scale and learn to classify NT1 and NT2 by identifying the most important features that are even not part of the current classification criteria such as the mean REM sleep latency of MSLT (which indicates no circular reasoning). These features are thus potential candidates for future classifications of narcolepsies. For validation reasons we decide to include NT1 although we are well aware that diagnosing NT1 is usually not a diagnostic challenge for clinicians. NT1 is especially suitable for validating our SGB model because all other forms of central hypersomnias are poorly defined and only NT1 is an accepted entity and could thus serve as a referenced “ground truth”, which is extremely important and crucial for validating the supervised learning model. In other words, humans can only trust that SGB is a suitable ML algorithm to correctly “learn” the clinical features of narcolepsy from the EU-NN database if it can successfully reveal the “ground truth” (i.e., classifying NT1 and NT2 correctly).

The performance of SGB depends on the characteristics of the database because boosting will fail to find a predictive model or give extremely poor performance if the database is too noisy (i.e., the updating of the weak learners may be over-emphasizing noisy examples)^14,15^. Therefore, the extremely high performance of our models under any combination of trees and tree depth (Fig. 1) could suggest good data quality of the EU-NN database, which will guarantee solid future analysis on this database. We find very few ‘misclassified’ predictions given by the model, as described in the Results section. Careful scrutiny of these cases, shows that these misclassifications are due to incorrect data interpretation by the physicians or false data entries instead of incorrect predictions by the model.

Our statistical analyses on the other predictors shown in Fig. 2 suggest that patients with NT1 have lower nocturnal PSG sleep efficiency and higher probability of carrying the HLA DQB1*06:02 subtype than patients with NT2, which is consistent with our current knowledge of narcolepsy^16,17^. The relative lower RI of these predictors compared to cataplexy features may be explained by the fact that they are not unique to the specific subtype of narcolepsy, i.e., patients with NT2 can also have lower PSG sleep efficiency and positive HLA DQB1*06:02, although the statistical differences between the subtypes are significant.

SGB identifies features with high contributions to the model, which are independent of existing diagnostic criteria and thus relatively unbiased by circular reasoning. It is worth to test the discriminative value and eligibility of these features for future diagnostic criteria. A clinical relevant example provided in this study is our analysis on the classification of NT1 and NT2 with mean REM sleep latency and the number of SOREMP of MSLT. While the number of SOREMP is an established marker for narcolepsy our SGB model gives a new ‘unexpected’ predictor, the mean REM sleep latency. This assumption is confirmed by results of several traditional statistical methods, suggesting that mean REM latency at MSLT will help to diagnose NT1 and NT2 in various clinical situations, such as for narcolepsy patients who present with atypical or rare cataplexy. In addition, our findings of correlations between mean REM sleep latency and hypocretin levels and its association to HLA DQB1*06:02 indicate biological links between the REM sleep propensity and the hypocretin and HLA systems. Another example for identifying new interesting parameters is the self-reported sleep length during weekdays and weekend. After removing parameters that are already included in the current diagnostic criteria our refined model provides high ranked subjective parameters such as the self-reported sleep length during weekend. Classical analysis confirms that NT2 patients sleep much longer on the weekends compared to NT1 patients, while their sleep lengths are similar during the week. These results may indicate that NT2 patients are capable to extend their sleep on the weekend to (partially) compensate their accumulated sleep debt during the week, whereas similar compensatory mechanisms may be not fully functional or not relevant in NT1 patients. These results may also indicate that NT2 are exposed to increased sleep pressure by life-style or circadian factors. Whether sleep extension in NT2 patients is a potential therapeutic approach to relieve narcolepsy symptoms or even a causal link to the pathophysiology of narcolepsy subtypes or a factor contributing to the progression of NT2 to NT1, are interesting topics calling for future studies.

Feature selection by SGB also helps us to better understand how single clinical aspect contributes to key features. Since multiple features of cataplexy are included and have different weights, the SGB output of relative influence is of special interest. During cataplexy attack, 74%, 62% and 53% NT1 patients report muscle weakness in face/jaw, neck/head drop, and whole body, respectively; while 42% and 51% patients report muscle twitches and slurred speech, respectively. These results suggest that muscle weakness, especially muscle weakness in face/jaw, is a more often symptom of cataplexy compared to muscle twitches and slurred speech. These results could explain why pattern of muscle weakness in face/jaw is ranked as No.1 predictor by SGB model and muscle twitches and slurred speech are ranked relative lower in cataplexy features.

Unfortunately our study has also weaknesses. First, the idea of the EU-NN database is to collect a systematic sample of all narcolepsy and patients with hypersomnias in the participating centers. For many reasons not all patients have been included from all centers. Although we assume that our sample gives a representative figure about European narcolepsy patients, a selection bias is possible. In addition, although our SGB model can fit the EU-NN database well with less overfitting (i.e., it shows good performance in the testing set), the generalization of our model to other narcolepsy databases such as the ones from the United States of America and China is unknown. We will know more in the future when the model is tested in other datasets.

Second, in our model the weight of CSF-hypocretin is quite small compared to the values of features of cataplexy. We are well aware that EDS together with low hypocretin is sufficient to diagnose NT1. Our results do not undervalue the importance of hypocretin in diagnosing NT1. Consistently, narcolepsy patients with hypocretin deficiency but no cataplexy are still predicted as NT1 by our machine. Actually, hypocretin level is ranked as No.1 in another model built without cataplexy features (see Fig. 4). The reason for the low weight of hyprocretin in the presented results is due mainly to absent data on hypocretin. Only 183 out of 598 NT1 patients have a confirmed hypocretin deficiency.

Third, the EU-NN database has missing values for key predictors other than hypocretin levels. Missing data is one of the common challenges of multicenter studies dealing with big dataset^18^. As mentioned in the Method section, only part of the dataset is mandatory due to feasibility/availability of assessment and sociocultural background among centers and countries. We check the missing values in other key predictors proposed by our model in Fig. 2 and find that 23.1% patients have missing value of HLA DQB1*06:02. The main reason of absent HLA DQB1*06:02 is because the test is costly and it is not included in current diagnostic criteria. 18% patients have missing values in PSG. In MSLT 14% patients have missing data for the number of SOREMP and the mean REM sleep latency, while another 28.5% patients have missing data only for mean REM sleep latency. There are multiple reasons for missing data in non-mandatory datasets such as human errors, dispensable tests for diagnostic purpose (e.g. when low CSF hypocretin levels already determine the diagnosis of NT1) to refusal of patients to perform a test, and variables not included in the current diagnostic criteria (e.g., we have more missing data in the mean MSLT REM latency compared to the number of SOREMP). It is usually difficult to test if the missing data are at random or not in multi-center studies but we recognize four lines of arguments indicating that missing data do not influence out results: First, after specifically checking the missing data we find them equally distributed as a common phenomenon in all of EU-NN centers and not related to single centers or countries. It is therefore unlikely that our results are systematically biased by sociocultural or regional factors. Second, missing data are not linked to specific age or gender either, indicating that patient factors may not bias our results. Third, although the key predictors have missing data, our model still successfully identifies the predictors as influential in classifying NT1/NT2. Fourth, we confirm the effect of predicting factors by using classical statistics, e.g., logistic regression, t-test, Fisher’s exact test. As these classical statistical analyses do not allow missing data we are forced to use only subgroups of patients with complete dataset. Since large number of missing data can reduce the weights of the predictors (e.g., the weight of hypocretin level is smaller than cataplexy features), it is thus reasonable to assume that the relative influences of these predictors are even larger without missing data.

Having these limitations in mind we recommend future well-controlled clinical and experimental studies to confirm stability and validity of mean REM sleep latency as a valuable candidate marker for classifying NT1 and NT2, such as evaluating the exact relationship between numbers of SOREMP and REM sleep latency (i.e. is a short latency of a single SOREMP episode specific enough to diagnose NT1?). Similarly, further comparisons between narcolepsy and other diagnosis with infrequent SOREMP episodes or other sleep-wake disorders causing EDS including circadian or respiratory sleep-wake disorders are needed to be taken into account before promoting mean REM sleep latency as a diagnostic marker for NT1. Considering the recent criticism on the number of SOREMP as instable and inconclusive diagnostic criteria^19,20^, for narcolepsy diagnosis alternative and/or additional parameters are desperately warranted.

## Conclusion

We used a mathematical model based on Stochastic Gradient Boosting (SGB) to find out, which kind of information can be derived from the complex European Narcolepsy Database. Our results show that machine learning approach may be valuable in diagnosing subtypes of narcolepsy and selecting new clinical features of narcolepsy on machine scale. It is important to test the validity and usefulness of our model in other datasets in near future. Future will then show whether clinicians could use modern computerized models in practice to enable more accurate diagnosis – and hence in planning best possible treatments for their patients.

## Methods

### Patients

Only validated patients, i.e., cases with complete mandatory data carrying an essential amount of information on the disease, are included in the analysis. The EU-NN has published definitions and standardized procedures for data acquisition. The rationale to define mandatory data is to cover essential clinical key aspects of hypersomnias that can be assessed in every center. In contrast non-mandatory data are expected to vary among different sleep centers due to sociocultural background of the population, available equipment or limited resources (e.g. CSF hypocretin levels).

Mandatory data contains:

- General information of the patients: e.g., birthday, sex, ethnic group, weight and height;
- Key symptoms: e.g., begin and duration of EDS, the frequency of episodes of irresistible daytime sleep, number and duration of scheduled naps, total daytime sleep time, presence of refreshing sleep episodes, first occurrence of cataplectic attacks, features and patterns of muscle weakness (whole body? partial face/jaw? partial neck/head drop? partial knees? partial arms/hands?), twitches, slurred speech, frequencies and duration of cataplexy attack, frequency and duration of sleep paralysis or hypnagogic hallucinations, subjective estimates upon awakening and during nocturnal sleep such as experiencing disturbed nocturnal sleep or sleep drunkenness in the morning, sleep length per night during the week and weekends, estimated sleep latency
- Medication: whether drug-native or using which medication currently;
- Clinical diagnosis according to International Classification of Sleep Disorders.
- Questionnaires, e.g. Epworth Sleepiness Scale (ESS)

In the majority of patients additional non-mandatory data are available and included in the current analysis. Non-mandatory data include sleep and vigilance test parameters from nocturnal polysomnography (PSG), multiple sleep latency test (MSLT), maintenance of wakefulness test (MWT), other vigilance tests, actigraphy, etc. Similarly, assessment of HLA tests from blood and CSF hypocretin levels are not mandatory but highly recommended items.

We include 702 narcolepsy patients (300 female, 402 male, mean age of the onset of EDS is 21.9 ± 10.9 (mean ± standard deviation), mean age of diagnosis is 32.2 ± 13.6, 598 patients are classified as NT1 and 104 patients are classified as NT2), within whom 183 patients show hypocretin deficiency. Each center of EU-NN has obtained ethical approval for publishing the patients’ data for scientific purpose by a national Institutional Review Board before entering patients (please refer to the section ‘Data access policy, ethics and security specifications’ of reference^1^). The scientific review committee of EU-NN has approved the study protocol. All methods are in accordance with the relevant guidelines and regulations. All patients have provided their informed consent to be entered into the EU-NN database and their data can be used for scientific studies.

### Stochastic Gradient Boosting (SGB)

The basic principle of boosting refers to the idea that a model constructed by week learners (e.g., model F_m_ built by m trees which are numbered from 1 to m) can be modified to become better by adding new tree, as the goal of each iteration step (i.e., generating a new tree) is to reduce the classification error made by current model. Therefore, after one iteration step a new model F_m+1_ is constructed by m + 1 trees (No. 1 to No. m + 1) and it corrects its predecessor F_m_. F_m+1_ is then boosted to model F_m+2_ in next iteration step and the consecutive error correction ultimately leads to a model providing the most accurate classification. The way of error reduction is arbitrary so that any loss function (loss function represents the inaccuracy of the prediction made by the machine learning algorithm during training, so machine can optimize its performance by minimizing the loss function) can be used depending on the problem being solved. In gradient boosting, a gradient descent procedure is used to minimize the loss function. Thus, the new constructed tree No. m + 1 is the one that minimized the loss function of model F_m_. The minimization is realized numerically by applying a steepest descent step that calculating the negative gradient of the loss function^7,10^.

**Figure 6:**
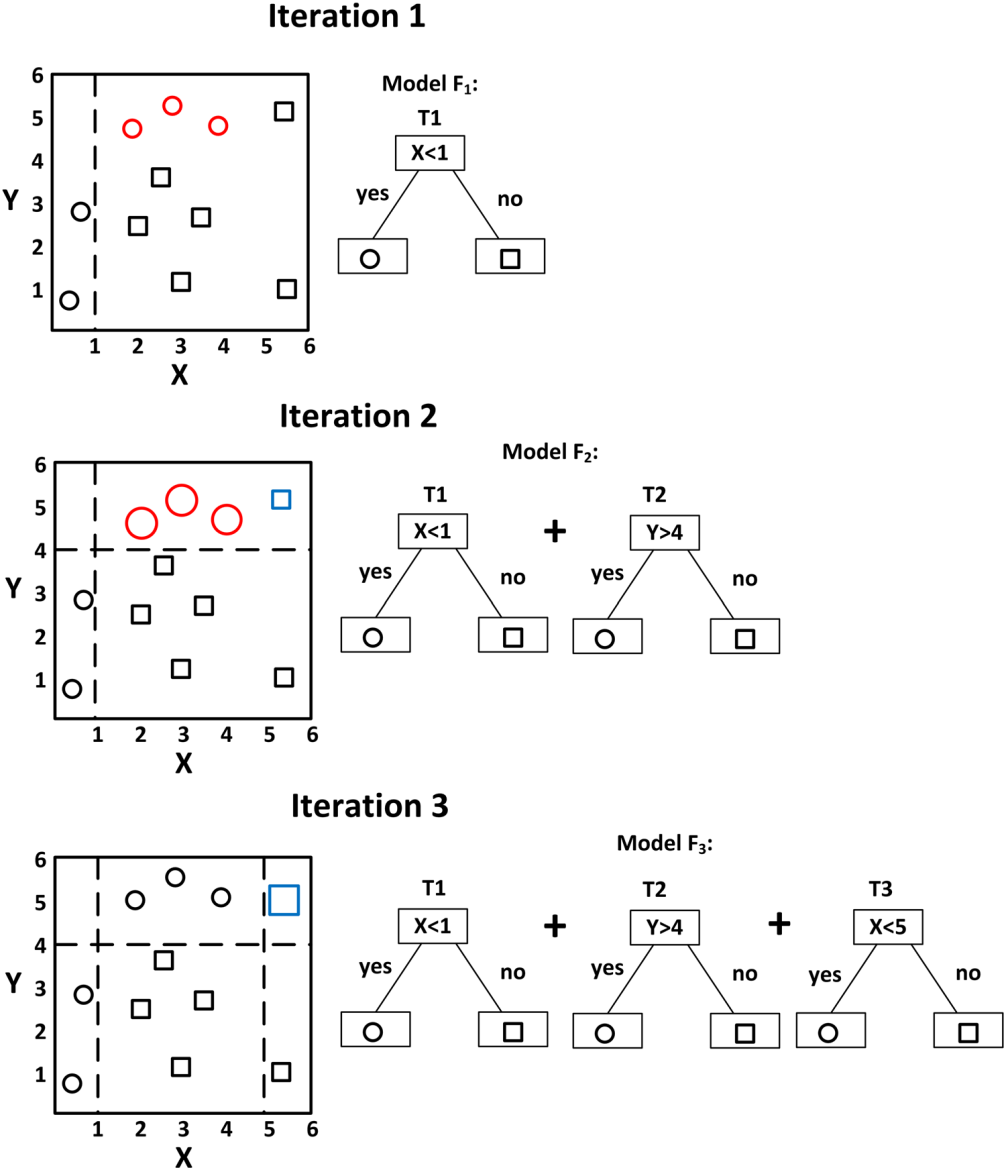
A simple example of visualizing gradient boosting.

A simple example of visualizing gradient boosting is illustrated in Fig. 6. The goal is to classify the circles and squares shown in the two-dimensional space. A decision stump (T1) generates a vertical line (let us assume X = 1) to classify the symbols. T1 is the simplest model F_1_ and symbols with X-axis smaller than 1 are classified as circles while values larger than 1 are classified as squares. We can see that model F_1_ has incorrectly classified three circles (marked in red colour) as squares. These three circles are the errors of model F_1._ In such case, higher weights are assigned to these three circles (i.e., they become bigger than other symbols) so that the second iteration can build another simple decision tree to correctly predict them. A horizontal line Y = 4 (i.e., decision stump T2) successfully separates the three red circles from most of the squares. The updated model F_2_ is the combination of the trees T1 and T2. We now see that one square marked in blue colour is misclassified by F_2_. The error of F_2_ has been reduced compared to one of F_1_. Next, the weights of the three red circles correctly classified by F_2_ are reduced to normal level, and the weight of the misclassified blue square is increased. Another vertical line X = 5 (i.e., decision stump T3) is generated during the third iteration step to separate the blue square from all the circles. The new model F_3_ is the sum of T1, T2 and T3 and it finally successfully classifies all the symbols. As a preliminary result Model F_3_ classifies these symbols quite well as compared to any of the individual trees.

Gradient boosting has the risk of overfitting. Overfitting indicates that precise predication can be drawn from the training set but the performance is low when using testing dataset (i.e., the trained model has poor generalization ability to predict new cases). This can be controlled by several regularization methods, including shrinkage and applying the SGB method. Previous studies find that to multiply a shrinkage factor *v* (0 < *v* < 1, which was also called ‘learning factor’) to the new build decision tree in each iteration step can dramatically improve the model’s generalization ability compared to the one without shrinking (i.e., overfitting is reduced)^7^. Besides the shrinkage strategy, SGB, a later developed algorithm based on gradient boosting, applies subsampling as a regulation technique to reduce overfitting^7,10^. At each iteration SGB samples a fraction (usually 50%) of the training data without replacement and uses these subsamples to grow the new tree^7^. Then the improvement of the prediction performance of the new model can be evaluated by predicting those subsamples which are not used in the building of the tree. Subsampling can reduce the computing time and produces a more accurate model^10^.

In SGB, the data preprocessing of centering and scaling are usually not required^7^. Predictors with near zero-variance (i.e., predictors that have one unique value or have very few unique values relative to the number of samples, or the ratio of the most frequent to the second most frequent value is large) are also allowed, although they usually have very minimal or zero contribution to the final prediction. For the sake of model complexity and computing time, we exclude children case specific predictors, near zero-variance predictors and the predictors with more than 80% missing values from further analysis. Finally 81 predictors are included to build the classifier. The data are randomly split into training and testing sets with a ratio of 75%:25%.

The SGB model is constructed with the caret package in R language^8^. The number of trees (i.e., the number of boosting iterations) and interaction depth (i.e., the depth of trees) are the two parameters tuned. The numbers of trees are set between 1000 and 4000 with a stepwise increment of 50, and the interaction depth is set to 1, 3, 5, 7 and 9, respectively. The shrinkage factor is fixed as 0.001. We select 10-fold cross-validation (CV) with 10 times repeats as the resampling scheme to estimate the test error.

### Statistical analysis

Receiver Operating Curves (ROC) are used as the performance metric to select the optimal model, i.e., the optimal tuning parameters are the ones constructing a model giving the largest value of the area under the ROC curve (AUC) in the resampling CV. Then we use the optimal model to predict the testing sets and the performances are evaluated with accuracy, AUC and Cohen’s kappa.

SGB model can provide the relative influence (RI) of each predictor contributing to the final predictive model. We use Wilcoxon rank sum tests and Welch two sample t-test to test if the medians and the mean of the effective continuous predictors are significantly different between NT1 and NT2 (P < 0.05). If the effective predictors contain count data, Fisher’s exact tests are used to evaluate if the odds ratio (OR) is statistically significant between NT1 and NT2 (P < 0.05). The data are expressed as mean ± standard error of mean (SE), unless otherwise stated. For the data that are not normally distributed the values are given as median (first quartile-third quartile). All statistical analyses are done using R.

### Data availability

The datasets generated during and/or analysed during the current study are available from the corresponding author on reasonable request.

## Electronic supplementary material

Supplementary Information
