# Supplementary material for: Exploring the clinical features of narcolepsy type 1 versus narcolepsy type 2 from European Narcolepsy Network database with machine learning
Source: Sci Rep. 2018 Jul 13;8:10628. doi: 10.1038/s41598-018-28840-w (PMC6045630; doi:10.1038/s41598-018-28840-w)
Supplement: Supplementary file 1 — Supplementary Information [file 41598_2018_28840_MOESM1_ESM.pdf]

Exploring the clinical features of narcolepsy type 1 versus narcolepsy type 2 from European Narcolepsy Network database with machine learning

Zhongxing Zhang [1] PhD, Geert Mayer [2] MD, Yves Dauvilliers [3] MD, PhD, Giuseppe Plazzi [4,5] MD, PhD, Fabio Pizza [4,5] MD, PhD, Rolf Fronczek [6] MD, PhD, Joan Santamaria [7] MD, Markku Partinen [8] MD, PhD, Sebastiaan Overeem [9,10] MD, PhD, Rosa Peraita-Adrados [11] MD, Antonio Martins da Silva [12] MD, Karel Sonka [13] MD, PhD, Rafael del Rio-Villegas [14] MD, Raphael Heinzer [15] MD, Aleksandra Wierzbicka [16] MD, Peter Young [17] MD, Birgit Högl [18] MD, Claudio L. Bassetti [20] MD, Mauro Manconi [19] MD, PhD, Eva Feketeova [21] MD, Johannes Mathis [20] MD, Teresa Paiva [22] MD, PhD, Francesca Canellas [23] MD, PhD, Michel Lecendreux [24, 25] MD, Christian R. Baumann [26] MD, Lucie Barateau[3] MD, Carole Pesenti[3] Msc, Elena Antelmi[4] PhD, Carles Gaig [7] MD, Alex Iranzo [7] MD, Laura Lillo-Triguero [11] MD, Pablo Medrano-Martínez [11] PhD, José Haba-Rubio [15] MD, Corina Gorban [1] MD, Gianina Luca [27] MD, PhD, Gert-Jan Lammers [6, 28] MD, PhD, Ramin Khatami [1,20]* MD

on behalf of the EUROPEAN NARCOLEPSY NETWORK (EU-NN)

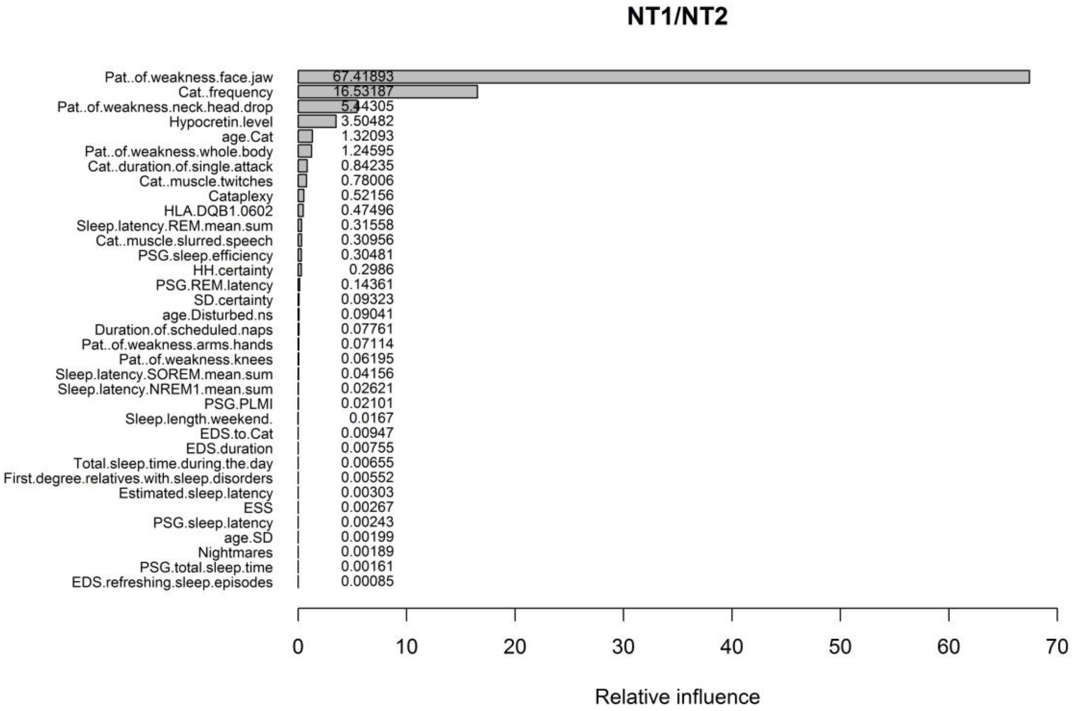

Figure 1. The relative influences of the 35 features contributing to the classification of NT1 and NT2 selected by our SGB model. The variable names written on the vertical axis. “Pat.” is short for pattern, “Cat.” is cataplexy. “Sleep.latency.REM.mean.sum” is the mean REM latency of multiple sleep latency tests. “HH.certainty” is the certainty of hypnagogic hallucinations. “PSG” is polysomnography, “SD.certainty” is sleep diagnosis certainty, “ns” is short for nocturnal sleep, “Sleep.latency.SOREM.mean.sum” is the number of SOREM (sleep onset REM) of multiple sleep latency tests, “Sleep.latency.NREM1.mean.sum” is the mean sleep latency of non-REM sleep stage 1 of multiple sleep latency tests, “PSG.PLMI” is the periodic limb movement index of PSG recordings, “EDS” is short for excessive daytime sleepiness, “EDS. to Cat.” is the time period (years) between showing EDS and cataplexy in patients, “ESS” is Epworth Sleepiness Scale, “age. SD” is the age of the patient diagnosed with sleep disorders.
